# Liquid-Liquid Phase Separation in Cancer Drug Resistance: Mechanisms and Therapeutic Opportunities

**DOI:** 10.32604/or.2026.076499

**Published:** 2026-05-21

**Authors:** Yiyang Zhao, Changchang Sun, Qihan Dong, Jiangyang He, Yan Wang, Ling Bi

**Affiliations:** 1 Department of Medical Oncology, Shuguang Hospital, Shanghai University of Traditional Chinese Medicine, Shanghai, China; 2 School of Natural Sciences, Faculty of Science and Engineering, Macquarie University, Sydney, NSW, Australia; 3 The Second Clinical Medical College of Guizhou University of Traditional Chinese Medicine, Guizhou, China

**Keywords:** Liquid-liquid phase separation, cancer drug resistance, biomolecular condensates, signaling pathways, targeted therapy

## Abstract

Liquid-liquid phase separation (LLPS) is an emerging biophysical principle that governs subcellular organization through the formation of dynamic, membraneless biomolecular condensates. This review aims to elucidate the multifaceted mechanisms by which dysregulated LLPS drives cancer drug resistance and to explore therapeutic strategies targeting oncogenic biomolecular condensates for improved anticancer outcomes. We synthesize evidence demonstrating that dysregulated LLPS drives cancer drug resistance through diverse mechanisms, including sustaining oncogenic transcription despite targeted therapies, creating physical barriers against chemotherapeutics, modulating immune checkpoint activity, enhancing DNA damage repair, promoting cancer stemness and radioresistance. By integrating insights from cell cycle control, cytoskeletal dynamics, and critical tumor signaling pathways, we highlight the pervasive role of LLPS in facilitating adaptive tumor responses. We also discuss how multi-omics approaches and advanced biophysical techniques facilitate studies of condensate dynamics and prognostic signature identification. Finally, we address the challenges and opportunities in developing therapeutic strategies that target oncogenic condensates, a promising approach to counteract resistance and improve anticancer treatments.

## 1 Introduction

**Figure 1:**
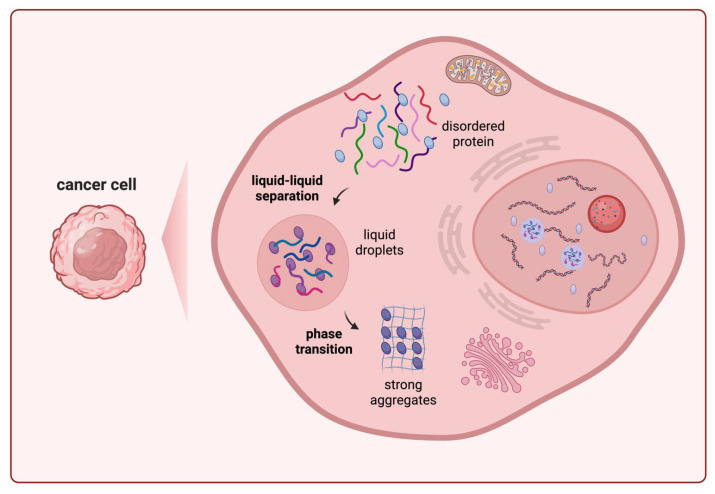
LLPS drives pro-tumorigenic processes through biomolecular condensates. Biomolecules with multivalent interactions and IDRs demix from the cytosol or nucleoplasm to form dynamic, membrane-less liquid droplets, which then transform into strong aggregates. These condensates (e.g., transcriptional hubs, stress granules) concentrate specific factors to drive pro-tumorigenic processes. LLPS: Liquid-liquid phase separation; IDRs: intrinsically disordered regions.

Liquid-liquid phase separation (LLPS) is an emerging biophysical principle governing subcellular organization through the formation of dynamic, membraneless biomolecular condensates. This process is driven by multivalent weak interactions, often mediated by intrinsically disordered regions (IDRs) within proteins [1], leading to the spontaneous segregation and concentration of proteins, nucleic acids, and other biomolecules from the cellular milieu. These liquid-like droplets exhibit properties such as rapid fusion, fluidity, and reversibility, enabling efficient compartmentalization without lipid barriers [2,3,4,5,6]. LLPS underpins the formation of membraneless organelles, including nucleoli, stress granules, and P-bodies. These condensates concentrate specific biomolecules to accelerate biochemical reactions while sequestering others for protection or storage [7,8]. In healthy cells, LLPS fine-tunes essential processes such as gene expression [9], signal transduction [10], and stress responses [11]. However, in cancer, factors such as oncogenic mutations, metabolic shifts, and hypoxic microenvironments dysregulate LLPS, leading to aberrant condensate formation that fuels tumor progression and therapy resistance (Fig. 1) [12,13].

A defining characteristic of LLPS is its capacity to enrich and sequester biomolecules. By concentrating specific molecules, LLPS can significantly enhance biochemical efficiency, such as accelerating oncogenic signaling transduction within condensates [14]. Conversely, by sequestering critical factors, it can offer protection under therapeutic stress, for instance, by pausing the translation of pro-apoptotic mRNAs in stress granules [15]. This mechanism also plays a role in cell cycle regulation by shielding critical components from damage. The ability of membraneless organelles to form and dissolve in response to cellular cues allows LLPS to exert precise spatiotemporal control over a wide array of fundamental cellular processes [16,17], with significant implications for disease pathogenesis [12]. 

Given the pervasive role of LLPS in cancer biology, its potential contribution to therapy failure is an area of intense interest. Drug resistance remains a major challenge in oncology, affecting most patients with metastatic cancers and often leading to relapse after initial treatment success [18]. We and others have found that LLPS can exacerbate this problem by facilitating the formation of localized hubs that shield oncogenic drivers, sequestering factors in stress granules, or amplifying pro-survival signals through super-enhancer condensates [19]. For instance, LLPS-mediated clustering of kinases in signaling pathways enhances pathway fidelity [20], while cytoskeletal condensates stabilize microtubules against depolymerizing agents like nocodazole [21]. Recent studies underscore LLPS’s centrality in resistance to targeted therapies, chemotherapy, immunotherapy, and radiotherapy, such as EGFR inhibitors in lung cancer, bortezomib and PD-1 blockers in multiple myeloma [22,23,24,25,26]. While previous reviews have highlighted the contribution of LLPS to core cancer hallmarks such as proliferation and immune evasion [27,28], the purpose of this review is to systematically examine the fundamental principles and multifaceted mechanisms by which dysregulated LLPS drives cancer drug resistance, integrating perspectives from cytodynamics (including cell cycle regulation, microtubule dynamics, cell migration, and apoptosis) with critical tumor-associated signaling pathways (such as cGAS-STING, PKA, Hippo/YAP, Wnt/β-catenin, and TGF-β). This objective aims to position LLPS as a targetable vulnerability, while discussing recent research advances and emerging therapeutic strategies to overcome resistance and refine anticancer treatments.

## 2 LLPS Contributions to Cancer Drug Resistance

LLPS fosters drug resistance by coordinating cytodynamic processes with major oncogenic signaling pathways [29]. These processes include cell-cycle control, microtubule dynamics, migration, and apoptosis [30]. This integration ultimately creates a highly adaptable and treatment-resistant tumor state [31]. Cytodynamics focuses on the movement, interaction, and transformation of biomolecules and organelles within cells, which are vital to fundamental cellular functions such as gene expression, protein folding, organelle localization, and responses to external signals [32]. By forming dynamic condensates, LLPS exerts broad influence over cell cycle control, microtubule stability, cell mechanics, and apoptosis [33]. These membraneless assemblies regulate gene expression and protein synthesis, guide organelle transport and positioning, and facilitate the cellular response to environmental cues [13]. Altogether, they govern cell growth, differentiation, and death, preserving cellular function and structural integrity [33]. LLPS regulates multiple tumor-related signaling pathways, making tumor cell signaling more selective and flexible by forming membraneless condensates to concentrate specific molecules or shield signaling proteins [34]. Studies have found that LLPS involves several key signaling pathways in tumor development, including the cGAS-STING pathway, PKA pathway, Hippo/YAP pathway, Wnt/β-catenin pathway and TGF-β pathway. These pathways are intertwined and jointly affect the occurrence and development of tumors [35,36]. In summary, LLPS endows tumor cells with adaptability to microenvironment changes through dynamic regulation of different signaling pathways. These pathways are interwoven, and LLPS regulates the concentration and activity of their signaling molecules with spatiotemporal precision [35,36]. This sophisticated control ultimately influences tumor growth and progression. This complex pathway network indicates that the role of LLPS in tumor biology is not only the regulation of a single pathway, but also lies at the core of multi-pathway synergies [37]. Below, we explore these intersections across key resistance modalities.

**Figure 2:**
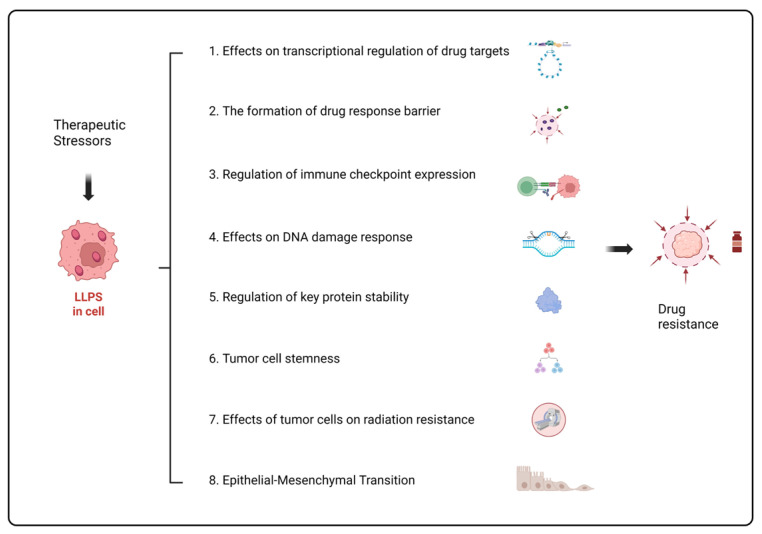
LLPS mechanisms in cancer drug resistance. The schematic summarizes key mechanisms through which LLPS fosters resistance, including: (1) sustaining oncogenic expression via transcriptional hubs; (2) creating physical/biochemical barriers against drugs; (3) modulating immune checkpoint expression; (4) enhancing DNA damage response; (5) stabilizing key oncoproteins; (6) promoting cancer stemness; (7) coordinating radioresistance; and (8) Epithelial-Mesenchymal Transition. LLPS: Liquid-liquid phase separation.

We propose that LLPS acts as a central adaptive hub which cancer cells deploy to coordinate a cohesive response to therapeutic assault. Diverse treatment stressors promote the formation of distinct yet interconnected biomolecular condensates. These condensates, in turn, orchestrate a network of resistance mechanisms (Fig. 2, Table 1).

### 2.1 LLPS in Transcriptional Regulation of Drug Targets

LLPS contributes to therapeutic resistance by forming transcriptional condensates that sustain oncogenic gene expression despite targeted inhibitors. The formation and function of cellular assemblies, including cytoskeletal structures, are often driven by weak, multivalent interactions among biomolecules, which are critical for numerous tasks [38,39]. The role of LLPS is evident in fundamental processes such as cell cycle regulation. For instance, in HeLa cells, phosphorylation of the IDR of Ki-67 during mitosis enhances its phase separation capacity [40]. While this study primarily elucidates the biophysical mechanism of chromosome dispersion, it is plausible to hypothesize that maintaining such high-fidelity mitotic machinery via LLPS could allow cancer cells to survive genomic stress induced by antimitotic chemotherapies. A growing body of research suggests that LLPS-driven biological processes form a fundamental defense layer against genotoxic therapies, including chemotherapy, in cancer cells. For example, transcription factors can drive osteosarcoma metastasis and chemotherapy resistance through LLPS-mediated aggregate formation [41]. The RNA helicase DDX6 regulates metabolic plasticity through phase separation, thereby conferring chemotherapy resistance to acute myeloid leukemia cells [42]. Furthermore, mitotic slippage, a typical drug resistance response, is also linked to unstable genomic processing and cellular reprogramming [43]. Therefore, the mechanism by which Ki-67 maintains mitotic fidelity through LLPS may provide a potential underlying basis for resistance to chemotherapeutic drugs targeting rapidly dividing cells.

**Figure 3:**
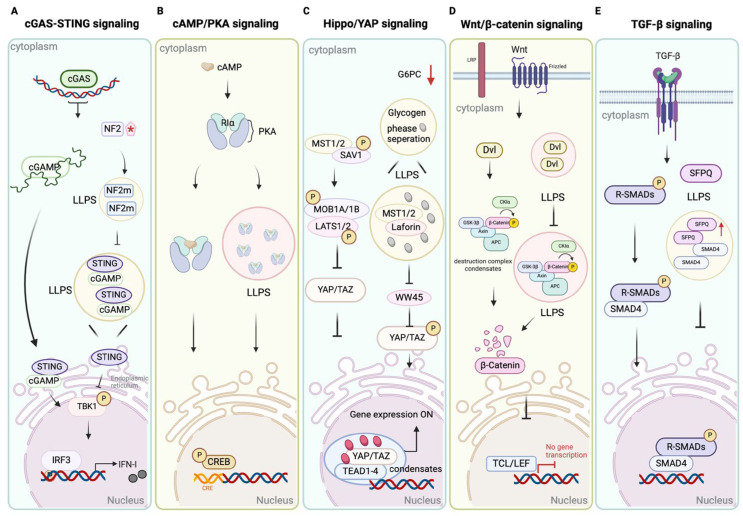
LLPS regulates core components of major cancer-associated signaling pathways. This schematic depicts how LLPS regulates five major tumor-associated pathways (cGAS-STING, cAMP/PKA, Hippo/YAP, Wnt/β-catenin, and TGF-β) through biomolecular condensate formation, enabling precise spatiotemporal control of signaling molecules. In cGAS-STING, LLPS suppresses (via NF2 mutations) immune signaling, contributing to immunotherapy resistance; in cAMP/PKA, RIα condensates compartmentalize cAMP for pro-survival signaling; in Hippo/YAP, LLPS inhibits kinase activity (e.g., via glycogen/Laforin) and sustains YAP/TAZ-TEAD transcription, driving resistance and stemness; in Wnt/β-catenin, LLPS modulates destruction complex assembly (via APC/Axin/Dvl), stabilizing β-catenin; in TGF-β, SFPQ condensates sequester Smads, shifting toward pro-tumorigenic outcomes including EMT. These interconnected regulations enhance tumor adaptability and therapy resistance across cancers. LLPS: Liquid-liquid phase separation; cGAS-STING: cyclic GMP-AMP synthase—stimulator of interferon genes; cAMP/PKA: cyclic adenosine monophosphate/protein kinase A; Hippo: hippopotamus; YAP: Yes-associated protein; Wnt: Wingless-related integration site; TGF-β: Transforming growth factor-beta; NF2: Neurofibromin 2; TAZ: Transcriptional coactivator with PDZ-binding motif; TEAD: TEA domain transcription factor; APC: Adenomatous polyposis coli; Axin: Axis inhibition protein; Dvl: Dishevelled; TCL/LEF: T-cell factor/lymphoid enhancer factor; SFPQ: splicing factor proline and glutamine-rich; Smads: Sma- and Mad-related proteins; EMT: Epithelial-Mesenchymal Transition.

Beyond cell cycle regulation, LLPS is a key mechanism in signal transduction pathways that influence drug response. The transcriptional co-regulator SGF29 forms LLPS-driven aggregates in multiple cell models, recruiting transcriptional machinery to induce p21Cip1 expression and promote senescence [44]. The Hippo/YAP pathway is another important signaling pathway regulated by LLPS (Fig. 3). YAP drives oncogenic transcription and chemoresistance in hepatoblastoma via liquid-liquid phase separation, while in lung cancer stem-like cells, it promotes stemness by directly binding OCT4 to induce SOX2 expression [45,46]. Furthermore, studies have shown that glycogen can encapsulate the Hippo pathway kinase Mst1/2 through interaction with Laforin, inhibiting its activity and activating YAP to promote tumor development [47]. Abnormal activation of this pathway can form a positive feedback loop against other inhibitory pathways, such as cGAS-STING and PKA (Fig. 3) [48,49]. Collectively, these findings demonstrate that LLPS regulates the Hippo/YAP pathway through diverse mechanisms-directly via YAP phase separation, indirectly through upstream modulators like glycogen, and via crosstalk with other key pathways—to coordinately drive oncogenic transcription, stemness, and therapy resistance.

Similarly, the Wnt/β-catenin pathway modulated by LLPS (Fig. 3). In colorectal cancer (CRC) cells, the IDRs of APC proteins drive LLPS to enhance Axin aggregation and accelerating β-catenin degradation [50]. Additionally, LLPS of Dishevelled 2 (Dvl2) can regulate the phase separation of Axin to mediate the assembly of the β-catenin degradation complex; this interaction can weaken the destruction complex and enhance Wnt signaling pathway activation, as demonstrated in HEK293T cells [51]. These opposing LLPS-mediated mechanisms on APC and Dvl2 therefore exert context-dependent effects on β-catenin stability, with APC LLPS helping to prevent its abnormal accumulation that drives tumorigenesis [52]. Components of the TGF-β pathway are likewise regulated by LLPS (Fig. 3). For instance, in liver cancer cells, the protein SFPQ can sequester or exclude Smad4 in phase-separated droplets, weakening its tumor-suppressive transcriptional activity [53]. Future studies may explore how LLPS-mediated changes in Smad localization, combined with crosstalk from Wnt/β-catenin and Hippo/YAP, LLPS-mediated changes in Smad localization may tilt TGF-β signals toward promoting tumor growth.

These mechanisms are associated with resistance against targeted therapies. In prostate cancer, LNCaP cells with AR F877L/T878A mutations exhibit sustained receptor activity under enzalutamide treatment, a process where enhanced LLPS plays a role [54]. Long non-coding RNAs (lncRNAs) can potentiate this process. In hepatocellular carcinoma (HCC) cells, knockdown of URB1-AS1 synergizes with sorafenib to increase ferroptosis and free iron levels [55], while in HCC models, knockdown of ZNF32-AS2 reduces proliferation and migration [56]. Multi-omics signatures have linked LLPS-related lncRNAs to prognosis and resistance in cancers such as prostate cancer [57]. Furthermore, the transcription factor FOXP1 forms nuclear condensates that regulate the super-enhancer of SP8, activating the homologous recombination repair (HRR) pathway to confer drug resistance, as demonstrated in small cell lung cancer [26]. These mechanisms in prostate cancer shows promise but are primarily from single studies using cell lines, replication in patient-derived xenografts is needed. Multi-omics signatures offer prognostic value but are limited by correlative data without causal validation [57].

The therapeutic potential of disrupting these condensates is emerging. For example, BRD4 Proteolysis-Targeting Chimeras (PROTACs) can disrupt oncogenic transcriptional hubs, re-sensitizing tumor to treatment [58]. These examples illustrate how LLPS-mediated transcriptional condensates, intertwined with cell cycle checkpoints and signaling cascades, enable tumor to evade targeted therapies. Disrupting condensate formation represents a promising strategy for restoring drug sensitivity.

### 2.2 LLPS-Mediated Barriers in Drug Response

Beyond transcriptional regulation, LLPS can erect physical and biochemical barriers that contribute to resistance, primarily through the precise spatial regulation of cell death pathways. For instance, in cellular models of undergoing TNF-induced necroptosis, PARP5A and RNF146 form condensates via LLPS, which recruit TAX1BP1 to accelerate the degradation of kinase-active RIPK1, thereby inhibiting necroptotic cell death [59]. Conversely, in HepG2 hepatocellular carcinoma cells under TGF-β stimulation, the LLPS of Smad2/3/4 complexes with TAT transcription factors enhances caspase-9 activity and promotes apoptosis [60]. External physical stimuli can also modulate apoptosis through LLPS. For example, exposure to static magnetic fields suppresses the LLPS of Tau-441, which reduces its recruitment of hexokinase (HK). This increases the availability of free HK to bind VDAC, competing with pro-apoptotic Bax, and ultimately diminishing Bax-driven apoptosis [61]. These examples collectively demonstrate the context-dependent precision with which LLPS governs apoptotic signaling. However these context-dependent effects, have not been widely replicated, and reliance on stimulation models such as TNF or TGF-β, may overlook tumor heterogeneity. Limitations include potential non-specific effects of magnetic fields [61] in biophysical assays.

Beyond apoptosis, LLPS creates signaling compartments that can be hijacked in cancer. The regulatory subunit RIα of protein kinase A (PKA) undergoes LLPS to form dynamic condensates enriched in active cAMP, enabling precise spatial control of cAMP/PKA signaling (Fig. 3) [62]. Disruption of RIα phase separation leads to aberrant cAMP signaling, which has been shown to increase proliferation and induce transformation in normal cells [63]. The compartmentalization of cAMP/PKA signaling through RIα LLPS likely exhibits crosstalk with other pathways, such as cGAS-STING, collectively shaping the tumor signaling network (Fig. 3).

LLPS also plays a direct role in multidrug resistance (MDR) mechanisms. In cancer cells, the cellular prion protein (PrP) undergoes LLPS, and intervention with melatonin disrupts this process, reducing drug efflux and reactive oxygen species (ROS) levels, as proposed in a review on MDR [64]. Similarly, in KMS11 myeloma cells, disruption of the SRC-3/NSD2 phase separation interface can increase sensitivity to bortezomib by downregulating anti-apoptotic proteins [23]. These findings highlight LLPS as a viable target for overcoming chemoresistance. PrP LLPS disruption by melatonin in cancer cells is intriguing but from a single study; replication *in vivo* is essential, as cell line models may exaggerate efflux mechanisms. Similarly, SRC-3 disruptors in myeloma [23] await confirmation in patient samples.

Collectively, these LLPS-mediated barriers protect cancer cells against chemotherapeutic agents like doxorubicin and vincristine. Therapeutic strategies are emerging to target these condensates. Melatonin, by targeting PrP LLPS, can restore drug sensitivity [64]. Additionally, small molecules that induce β-catenin LLPS have been shown to suppress tumor growth and invasion [65]. By integrating apoptotic evasion, migratory adaptation, and signaling isolation, LLPS forms multifaceted barriers that sustain multidrug resistance. Disrupting these protective condensates represents a promising approach to enhance the efficacy of conventional chemotherapy across diverse cancer types.

### 2.3 LLPS Regulation of Immune Checkpoint Expression

LLPS plays a critical role in modulating immune checkpoint expression, enabling tumor immune evasion through mechanisms that intersect with innate immunity signaling. A key pathway involved is the cGAS-STING pathway, which plays an important role in anti-tumor immunity (Fig. 3) [66]. Specifically, upon recognizing cytoplasmic DNA, cGAS generates cGAMP to activate STING, triggering the production of type I interferons and inflammatory factors to potentiate the innate immune response [67]. Notably, in the presence of abundant DNA, cGAS can form active condensates via LLPS, significantly amplifying the intensity of this immune response [68,69]. Recent studies have extended this to specific cancers; for instance, in breast cancer models, LLPS-mediated cGAS condensates enhance STING activation, promoting interferon responses that can be hijacked by tumors to upregulate PD-L1 and foster resistance to immunotherapy [70,71]. Conversely, the tumor suppressor NF2 (Merlin) can modulate this process. Mutations in the FERM domain of NF2 enable it to form aggregates that abolish TBK1 activation, thereby suppressing immune signaling (Fig. 3) [72]. This dual role illustrates that LLPS not only enhances immune responses but may also participate in a complex balancing mechanism to adapt to the tumor microenvironment.

Recent studies further highlight that LLPS influences immune evasion by forming condensates that sequester immune effectors. Targeting these condensates can restore T-cell infiltration and improve response to immunotherapy [73]. Furthermore, the Hippo/YAP pathway contributes to immune resistance. In lung cancer models under anti-PD-1 treatment, YAP inhibitors can overcome resistance by disrupting YAP-mediated LLPS [74]. 

Besides, in head and neck squamous cell carcinoma (HNSCC), multi-omics analyses reveal LLPS-related subtypes that upregulate both PD-L1 and CTLA-4, correlating with poorer immunotherapy outcomes and highlighting spatial heterogeneity in checkpoint expression [75]. Emerging studies indicate that LLPS-driven mechanisms are involved in regulating the expression of immune checkpoint proteins like PD-L1, thereby contributing to broader immune resistance mechanisms within the tumor microenvironment. This is because PD-L1 expression is critically regulated at the nexus of RNA metabolism and immunosuppression, a process where the formation of biomolecular condensates via LLPS plays an essential role in its transcriptional and post-transcriptional control [24].

cGAS condensate amplification [76,77] has been replicated in immune models but less so in tumors; a limitation is the DNA-abundance dependency, which may vary in hypoxic microenvironments. YAP-mediated resistance in lung cancer [74] is promising but requires broader validation beyond anti-PD-1 models. Also, these mechanisms, which involve key pathways such as cGAS-STING and Hippo/YAP, demonstrate how LLPS enables tumor to evade immune surveillance. Therapeutically, combining LLPS disruptors with checkpoint inhibitors shows promise [73]; for example, targeting cGAS-STING condensates in immunotherapy-resistant tumor enhances anti-PD-1 efficacy by boosting innate immune activation and reducing checkpoint expression [78]. This approach could address resistance in diverse cancers, and LLPS-based subtyping has been shown to predict immunotherapy response in cancers like HNSCC [75]. Thus, combining conventional immunotherapy with agents that disrupt specific condensates represents a promising strategy to boost antitumor immune responses.

### 2.4 LLPS in Coordinating Cellular Stability and DNA Damage Response (DDR)

LLPS plays a critical role in maintaining cellular stability and promoting treatment resistance, notably by coordinating microtubule-driven functions and facilitating the recruitment of DNA repair machinery. LLPS influences cancer cell migration by regulating cytoskeletal dynamics and cell adhesion. Cell migration relies on coordinated cytoskeletal remodeling and adhesive contacts [79]. LLPS contributes to this process by forming dynamic condensates that affect both mechanical forces and signaling. For example, in HCT116 CRC cells, overexpression of SNRPD2 disrupts the LLPS of PABPN1, which in turn alters the alternative polyadenylation (APA) of CTNNBIP1, thereby boosting cell migration [80]. In focal adhesions, the LIM-domain protein LIMD1 undergoes LLPS to influence adhesion maturation and mechanical signaling, regulating cell spreading and contraction on the extracellular matrix [81]. LLPS is equally critical for maintaining cell-cell junctions. The tight junction protein zonula occludens (ZO) forms membrane-adjacent condensates that selectively recruit junctional proteins, facilitating rapid junction assembly, as demonstrated in MDCK cells with ZO mutants [82]. These processes provide a framework for how LLPS coordinates migration and morphological changes.

The regulation of microtubule dynamics is a key function of LLPS. As core components of the cytoskeleton, microtubules maintain cell shape, support division, and enable intracellular transport through precisely regulated polymerization dynamics [38,39]. Evidence indicates that LLPS significantly influences these processes [21]. A prominent example is mitotic spindle assembly. The spindle apparatus is essential for accurate chromosome segregation [83], and proteins such as nuclear mitotic apparatus protein (NuMA) undergo LLPS to form liquid-like droplets that travel along microtubules to the spindle poles, thereby regulating microtubule flux, spindle length, and overall architectural stability [84,85]. Similarly, the protein TPX2 promotes microtubule nucleation and growth through LLPS, forming membraneless compartments on the microtubule surface that enhance polymerization efficiency [86]. This principle extends to other microtubule-associated proteins. For instance, LLPS-driven condensates of CLIP-170 and EB3 can accelerate microtubule growth and suppress depolymerization [87,88]. Likewise, the microtubule-stabilizing protein Tau undergoes LLPS to form high-concentration clusters, which strengthen its binding to microtubules and stabilize the polymer lattice [89,90]. Overall, LLPS contributes to multiple aspects of microtubule organization, from regulating dynamics to localizing tubulin, thereby enhancing the resilience and functional efficiency of the microtubule network and ensuring accurate chromosome segregation [91]. The role of Ki-67 in forming a protective condensate that ensures mitotic fidelity after damage further underscores the link between LLPS and genome stability [92]. TPX2 and NuMA LLPS effects have partial replication in spindle models but need *in vivo* tumor confirmation; *in vitro* polymerization assays may not capture full cytoskeletal interactions.

Beyond maintaining structural integrity, LLPS is centrally involved in the DDR by facilitating the rapid recruitment and concentration of repair factors at damage sites [93,94]. For example, the BRCA1/BARD1 complex, a key complex in homologous recombination repair, undergoes LLPS to form subnuclear condensates, a process potentially underpinning its recruitment to DNA double-strand breaks. Consequently, siRNA-mediated depletion of BRCA1 in U2OS cells significantly impairs DNA repair efficiency [95]. The DNA sensing cGAS-STING pathway, which forms active condensates in response to cytoplasmic DNA (Fig. 3) [68,69], also exhibits crosstalk with the DDR, contributing to broader genomic surveillance [66,67,68,69]. Furthermore, specific signaling axes are regulated through LLPS to promote resistance. In HCT116 colon cancer cells, knockdown of SENP1 reduces the formation of repair-related condensates, increasing chemosensitivity. This aligns with findings that the SENP1-RNF168 axis drives resistance in colon cancer [96]. Transcriptional recovery after damage is also modulated by LLPS. It has been demonstrated that upon DNA damage, activated PARP1 binds to and PARylates the transcription elongation factor P-TEFb, which disrupts its LLPS and consequently inhibits the release of RNA polymerase II from promoter-proximal pausing, thereby suppressing global transcription [97]. In summary, LLPS plays a multidimensional and critical role in the DNA damage response by regulating complexes or pathways. However, these mechanistic insights are largely derived from studies in specific cell models. The generality of these conclusions, particularly their functional relevance across pan-cancer contexts, remains to be systematically validated in future research.

Building on these mechanistic insights, the translational relevance of LLPS in oncology is increasingly evident. Pan-cancer analyses indicate that LLPS-related signatures can predict defects in the DDR [75]. Therapeutically, targeting the formation of DNA repair condensates represents a promising strategy. For instance, inhibiting the formation of BRCA1 foci—a process potentially driven by liquid-liquid phase separation—by compromising CDK1 activity, has been shown to sensitize BRCA-proficient tumor cells to PARP inhibitors [98,99]. Integrating the roles of LLPS in microtubule stability and DNA repair underscores its comprehensive contribution to chemo- and radioresistance [21,98]. For example, in CRC, decreased LLPS of RNF168, mediated by SENP1, promotes DNA damage repair and is associated with drug resistance [96]. This suggests that developing therapies targeting the IDRs of these proteins could improve treatment outcomes in brain tumor and other malignancies.

### 2.5 LLPS Regulation of Key Protein Stability

LLPS contributes to cancer progression and therapy resistance by modulating the stability of key oncoproteins and tumor suppressors, thereby orchestrating an integrated network of control over apoptosis and signaling pathways. A prominent example involves the SPOP-Cul3-RBX1 E3 ubiquitin ligase complex, where cancer-associated mutations impair its normal phase separation, reducing the degradation of oncogenic substrates and promoting tumorigenesis, as demonstrated in prostate cancer models [100].

Signaling pathways are critically regulated through LLPS-mediated control of protein stability (Fig. 3). In the PKA pathway, the regulatory subunit RIα undergoes LLPS to form condensates that sequester active cAMP, facilitating localized signaling; the disruption of RIα phase separation can lead to unbalanced cAMP/PKA signaling that promotes tumor cell proliferation and transformation [63]. Similarly, within the Wnt/β-catenin pathway, the scaffold proteins APC and Axin form LLPS-driven condensates that promote the degradation of β-catenin, while the phase separation of the signalosome protein Dvl2 can antagonize the formation of this β-catenin destruction complex, thereby enhancing Wnt pathway activity [50,51,52]. LLPS also directly regulates cell death pathways, as evidenced by the finding that the E3 ligase RNF146 forms phase-separated condensates which promote the degradation of kinase-active RIPK1, thereby inhibiting necroptotic cell death [59].

The influence of LLPS extends to the cellular response to chemotherapeutic agents and inspires novel therapeutic strategies. For instance, the tumor suppressor SPOP normally forms phase-separated nuclear compartments that are essential for its E3 ubiquitin ligase activity; cancer-associated mutations disrupt this LLPS-driven assembly, leading to oncogenic substrate accumulation and presenting a potential target for therapeutic intervention [100]. In prostate cancer, SPOP mutations enhance the liquid-liquid phase separation capacity of p62/SQSTM1, which in turn promotes p62-dependent autophagy and activation of the antioxidant master regulator Nrf2, contributing to cancer cell survival [101]. More broadly, oncogenic fusion proteins frequently exploit LLPS to drive therapeutic resistance. In acute promyelocytic leukemia (APL), the PML-RARα fusion protein undergoes aberrant LLPS, forming microspeckles that co-assemble with the transcriptional coactivator BRD4. This process redistributes BRD4 to oncogenic super-enhancers, stabilizing a pro-leukemic transcriptional program essential for APL survival [102]. Furthermore, neddylation-induced aberrant phase separation of PML-RARα disrupts the assembly of functional PML nuclear bodies, promoting leukemogenesis [103]. These findings collectively suggest that disrupting the phase separation of such oncogenic condensates is a promising therapeutic avenue. It is noted that while the impact of SPOP mutations has been replicated in prostate models, broader validation across cancer types is awaited. Strategies like PROTACs show conceptual promise for targeting condensates but face challenges such as off-target risks *in vivo*. 

In summary, by forming condensates that stabilize oncoproteins or destabilize tumor suppressors at the intersection of apoptosis and key signaling pathways such as PKA and Wnt, LLPS helps sustain oncogenic drivers (Fig. 3). These findings highlight the value of developing targeted degradation strategies, including PROTACs, to overcome treatment resistance in cancers where LLPS undermines protein homeostasis.

### 2.6 LLPS and Tumor Cell Stemness

LLPS sustains tumor cell stemness by enabling cancer stem cells (CSCs) to evade cell cycle arrest and apoptosis, thereby contributing to therapy resistance and relapse [27]. This is primarily achieved through the formation of dynamic transcriptional condensates that activate and maintain the stemness program [104]. 

A central mechanism involves the LLPS-driven formation of stemness-associated transcriptional hubs. In breast CSCs, for example, the TAZ transcription factor undergoes phase separation and co-condenses with NANOG on stiff matrices, leading to the upregulation of core pluripotency factors SOX2 and OCT4 [104]. Similarly, in liver cells, glycogen overload-induced inhibition of laforin promotes aberrant LLPS of YAP, which in turn activates oncogenic gene expression and tumorigenesis [47]. The Hippo/YAP pathway is particularly notable, as YAP itself can form transcriptional condensates through LLPS, significantly amplifying the expression of stemness-related genes (Fig. 3) [105,106].

Beyond direct transcriptional regulation, LLPS also influences cell behavioral programs that are critical for CSCs, such as migration and niche engagement. In fibroblasts, the protein LIMD1 forms LLPS-based condensates to regulate durotaxis, and its loss impairs migration speed on stiff substrates [81]. This highlights how LLPS integrates biophysical cues with cell behavioral programs relevant to CSCs dissemination and niche engagement.

Building upon the mechanisms by which LLPS sustains CSC stemness, the translational implications of targeting these condensates are increasingly supported by experimental evidence. The most direct evidence comes from the pharmacological disruption of oncogenic condensates. For instance, in preclinical breast cancer models, disrupting the phase-separated condensation of TAZ with NANOG has been proven to significantly reduce the CSC population and suppress tumor recurrence, providing a proof-of-concept for directly targeting stemness-associated transcriptional condensates [104]. Furthermore, multi-omics analyses indicate that LLPS-related molecular signatures hold significant prognostic value. For example, a model constructed based on LLPS-related lncRNAs has been shown to effectively predict the risk of biochemical recurrence in prostate cancer patients [57], suggesting a potential association between these signatures and disease (including stemness-associated) relapse. However, current research also faces certain limitations. The key finding regarding TAZ phase separation in breast CSCs, for instance, is primarily based on a single study system, and the generalizability of this mechanism across diverse CSC populations requires further validation. Additionally, *in vitro* models focusing on biomechanical regulation may not fully recapitulate the complex tumor microenvironment *in vivo*, particularly overlooking critical factors such as immune interactions. Therefore, future efforts should focus on validating these targeting strategies more broadly across various cancer types and within more complex *in vivo* models to advance their clinical translation.

Collectively, these findings establish that LLPS enables a subset of CSCs to maintain stem-like traits, thereby enhancing their resilience to conventional treatments. This mechanistic insight is exemplified in breast cancer, where TAZ-NANOG phase separation maintains stemness and confers chemoresistance. Notably, increased matrix stiffness enhances this LLPS process, suggesting that targeting the biomechanical microenvironment could synergize with condensate-disrupting therapies [104]. Ultimately, disrupting LLPS in CSCs represents a promising strategy to target the root of tumor recurrence and overcome drug resistance.

### 2.7 LLPS Effects on Radioresistance

LLPS is proposed to enhances tumor radioresistance by orchestrating a synergistic response that integrates DNA damage repair with the stabilization of cytoskeletal structures [21,98]. This coordination is critical for cellular survival following radiation-induced damage [107]. Evidence from CRC cells indicates that siRNA-mediated knockdown of NOP53, a protein involved in ribosome biogenesis and stress response, reduces radioresistance and suppresses cell growth, highlighting its functional role in this process [107]. However, this finding awaits independent replication in other models or studies.

The ability of LLPS to modulate key nuclear processes after radiation exposure is a key mechanism. In nasopharyngeal carcinoma cells, radiation induces the PARylation of Cyclin T1 (CycT1), a component of the positive transcription elongation factor b (P-TEFb) complex. This modification disrupts P-TEFb LLPS, leading to prolonged RNA polymerase II pausing and a significant extension of transcriptional arrest, which is part of the cellular stress adaptation [108]. However, the functional significance of radiation-induced PARylation *in vivo*, particularly within tumor microenvironments, warrants further investigation using models such as xenografts.

Concurrently, LLPS is hypothesized to contribute to radioresistance by maintaining cytoskeletal integrity and mitotic fidelity. *In vitro* microtubule polymerization assays demonstrate that disruptors of the microtubule-plus-end binding proteins EB1 and CLIP-170, which undergo LLPS to promote microtubule growth, can increase the microtubule growth rate [88]. Furthermore, at the mitotic spindle poles, the protein NuMA utilizes LLPS to form stable compartments that ensure proper spindle architecture and function [84]. These mechanisms, crucial for cell division, could underpin the survival and proliferation of cancer cells following radiation treatment.

These processes are closely linked to the activation of innate immune signaling pathways in response to radiation-induced DNA damage. The cGAS-STING pathway, which forms active condensates upon cytoplasmic DNA sensing, is a crucial sensor for radiation-induced micronuclei, triggering type I interferon responses that can influence tumor cell survival and the tumor microenvironment (Fig. 3) [66].

Therapeutically, the concept of biomolecular condensates as drug reservoirs presents a novel strategy for enhancing cancer therapy [109]. The finding that disrupting NOP53 LLPS heightens radiation sensitivity underscores the potential of targeting specific condensates [107]. In summary, by concurrently stabilizing microtubule homeostasis and enhancing DNA repair, LLPS may confer a robust survival advantage to tumor following radiation, positioning it as a compelling therapeutic target for radio-sensitization.

### 2.8 LLPS in Epithelial-Mesenchymal Transition (EMT) and Therapy Resistance

EMT is a dynamic process whereby cancer cells lose epithelial characteristics and gain mesenchymal traits, facilitating invasion, metastasis, and acquisition of therapy resistance [110]. Emerging evidence highlights LLPS as a key regulator of EMT, where biomolecular condensates orchestrate the spatiotemporal control of EMT transcription factors and signaling pathways. For instance, the oncogenic transcriptional coactivators YAP/TAZ can form pathogenic condensates via LLPS, which drives the expression of genes associated with EMT, stemness, and therapy resistance across multiple cancer types. This phase separation-mediated amplification of oncogenic signaling contributes to resistance against various therapies [111].

Furthermore, the TGF-β pathway, a canonical EMT driver, is modulated by LLPS. In hepatocellular carcinoma cells, LLPS-driven condensates of Smad complexes with co-factors like SFPQ sequester tumor-suppressive signals, tilting TGF-β toward pro-EMT and drug-resistant phenotypes (Fig. 3) [53]. LLPS drives EMT programs by compartmentalizing and activating key transcription factors and signaling components. This aberrant condensation not only promotes the mesenchymal transition but also establishes a barrier to therapy by enriching pro-survival factors and altering drug sensitivity [112].

Therapeutically, disrupting EMT-related condensates offers promise. For example, inhibitors targeting YAP LLPS have reversed EMT in preclinical models, re-sensitizing tumors to chemotherapy and immunotherapy [110]. However, evidence is primarily *in vitro* and awaits broader *in vivo* and clinical validation to address context dependence, such as microenvironmental cues such as hypoxia, stiffness. Integrating this with multi-omics signatures could identify EMT-LLPS biomarkers for personalized therapy.

Collectively, the evidence discussed in this section supports a model wherein LLPS functions as a central organizing hub for drug resistance. Rather than operating in isolation, the mechanisms of transcriptional sustainment, barrier formation, immune evasion, DNA repair, protein stabilization, stemness maintenance, and radioresistance are coordinated through the dynamic and context-dependent assembly of biomolecular condensates (Fig. 2, Table 1). This framework explains how tumor can mount a unified adaptive response: for example, DNA damage-induced condensates can simultaneously enhance repair and influence transcription, while cytoskeletal condensates can form physical barriers and promote migratory adaptation. Viewing LLPS as this central integrator provides a more holistic understanding of tumor adaptability and underscores the therapeutic potential of disrupting the hub itself.

To facilitate comparison, Table 1 summarizes the key LLPS-mediated mechanisms, highlighting evidence strength and limitations.

**Table 1:** Summary of LLPS-mediated drug resistance mechanisms.

Mechanism	Type of Evidence	Cancer Type	Context	Major Features
Transcriptional Regulation of Drug Targets (2.1)	Primarily *in vitro* (e.g., HeLa, HEK293T cells); some *in vivo* (xenografts)	Prostate cancer, hepatocellular carcinoma, lung cancer	Oncogenic mutations (e.g., AR F877L), lncRNAs, pathway activation (Hippo/YAP)	Forms condensates sustaining oncogenic expression; integrates with cell cycle and signaling (e.g., Wnt/β-catenin)
LLPS-Mediated Barriers in Drug Response (2.2)	*In vitro* (e.g., HepG2 cells); limited *in vivo*	multiple myeloma, hepatocellular carcinoma	Stress stimuli (e.g., TNF, TGF-β), external physical stimuli (e.g., magnetic fields)	Establishes physical/biochemical barriers; regulates apoptosis and signaling (e.g., PKA) via condensates
Regulation of Immune Checkpoint Expression (2.3)	*In vitro* (e.g., breast cancer models cells); emerging *in vivo*/clinical correlations	Breast cancer, lung cancer, head and neck squamous cell carcinoma	IFN-γ stimulation, pathway mutations (e.g., NF2)	Modulates PD-L1/CTLA-4 via condensates; intersects cGAS-STING and Hippo/YAP for immune evasion
Coordinating Cellular Stability and DDR (2.4)	*In vitro* (e.g., U2OS, HCT116 cells); some *in vivo* (xenografts)	Colorectal cancer, glioblastoma	DNA damage, microtubule stressors	Recruits repair factors; stabilizes microtubules via condensates (e.g., TPX2, NuMA)
Regulation of Key Protein Stability (2.5)	*In vitro* (e.g., prostate models); limited *in vivo*	Prostate cancer, leukemia	Mutations (e.g., SPOP), fusion proteins	Stabilizes oncoproteins via condensates; crosstalk with PKA/Wnt pathways
Tumor Cell Stemness (2.6)	*In vitro* (e.g., breast CSCs); *in vivo* (xenografts)	Breast cancer, hepatocellular carcinoma, pancreatic cancer	Matrix stiffness, glycogen overload	Maintains stemness hubs (e.g., TAZ/YAP condensates); promotes relapse
Radioresistance (2.7)	*In vitro* (e.g., CRC, nasopharyngeal cells); awaits broader *in vivo*/clinical	Colorectal cancer, nasopharyngeal carcinoma	Radiation exposure, DNA damage	Coordinates repair and cytoskeleton; forms condensates post-radiation (e.g., NOP53)
EMT Regulation (2.8)	Primarily *in vitro* (e.g., breast, lung cancer models); emerging preclinical reviews, limited *in vivo*	Breast cancer, lung cancer, hepatocellular carcinoma, prostate cancer	TGF-β stimulation, matrix stiffness, Hippo/YAP activation	Forms condensate with EMT factors (e.g., YAP/TAZ, Snail, Smad); promotes tumor plasticity, invasion, and multi-drug resistance via pathway crosstalk

Note: LLPS: Liquid-liquid phase separation; Hippo/YAP: Hippo/Yes-associated protein; Wnt: Wingless-related integration site; TNF: Tumor Necrosis Factor; TGF-β: Transforming growth factor-beta; PKA: protein kinase A; IFN-γ: Interferon-gamma; PD-L1/CTLA-4: Programmed Death-Ligand 1/Cytotoxic T-Lymphocyte-Associated Protein 4; cGAS-STING: cyclic GMP-AMP synthase—stimulator of interferon genes; NF2: Neurofibromin 2; DDR: DNA Damage Response; TPX2: Targeting Protein for Xklp2; NuMA: nuclear mitotic apparatus protein; SPOP: Speckle-type POZ protein; CSCs: cancer stem cells; TAZ/YAP: Transcriptional coactivator with PDZ-binding motif/Yes-associated protein; CRC: colorectal cancer; NOP53: Nucleolar protein 53; EMT: Epithelial-Mesenchymal Transition; Smad: Sma- and Mad-related protein.

### 2.9 Current Limitations and Future Directions in LLPS-Mediated Drug Resistance Research

Despite providing a novel perspective on cancer drug resistance, the application of LLPS knowledge faces several limitations. These include the heavy reliance on *in vitro* and cell line models that may not recapitulate the tumor microenvironment, the challenge of achieving specificity in LLPS interventions without off-target effects, the lack of standardized quantitative metrics for condensate properties, and the scarcity of clinical correlations linking LLPS signatures to patient outcomes.

The application of LLPS in oncology continues to face significant challenges. Key diagnostic hurdles include leveraging condensate features in clinical samples, such as liquid biopsies, as biomarkers for predicting drug resistance. Targeting challenges involve distinguishing and selectively disrupting pathological condensates while preserving the function of physiological ones. Technical obstacles center on developing imaging technologies for real-time, dynamic monitoring of condensate formation and dissolution in live animal models.

To address these challenges, research on LLPS in drug resistance needs to prioritize several directions. These include prioritizing *in vivo* validation using patient-derived organoids or xenografts, developing selective inhibitors targeting cancer-specific IDRs, and establishing unified biophysical assays for condensate quantification. Integrating AI-driven modeling of phase diagrams with multi-omics could accelerate identification of druggable interfaces, ultimately facilitating clinical trials for LLPS-targeted therapies to reverse resistance. Technologically, it is also important to optimize detection technology, such as super-resolution imaging, develop more accurate *in vitro* models, and establish standardized quantitative analysis methods. In addition, combining multi-omics technologies, such as proteomics and single-cell sequencing, may help elucidate the comprehensive role of LLPS in tumor and advance the clinical translation of targeted therapeutic strategies.

## 3 Advances in LLPS Research Techniques

Methodological innovations expand our ability to probe the mechanisms and functional consequences of LLPS in biological systems. A diverse and complementary toolkit of techniques has been established to characterize the formation, material properties, and regulatory dynamics of biomolecular condensates. Key approaches include FRAP for analyzing condensate fluidity [40], *in vitro* reconstitution assays for defining minimal components and interactions [40,113], optogenetic platforms for spatiotemporal control of condensation [114], nuclear magnetic resonance (NMR) spectroscopy for resolving atomic-level structural features [113], and PROTACs for interrogating condensate function through targeted protein degradation [58]. The integrated application of these methods, as exemplified by studies of Ki-67 in mitotic regulation [40] and YAP in transcriptional activation [105], provides multifaceted insights into LLPS dynamics.

### 3.1 Fluorescence Recovery after Photobleaching

FRAP is widely employed to confirm the dynamic nature of LLPS by bleaching fluorescently tagged components and tracking fluorescence recovery to assess molecular mobility [115]. This approach reveals essential properties of biomolecular condensates, including their fluidity and exchange rates, offering critical insights into how phase-separated structures form and function [116]. While powerful for investigating droplet reversibility and fluid characteristics, FRAP is less suited to resolving smaller-scale molecular interactions.

### 3.2 In Vitro Reconstitution

*In vitro* reconstitution experiments recapitulate LLPS using purified components under controlled biochemical conditions, which can be modulated by factors such as ionic strength and pH [117]. This bottom-up approach is invaluable for elucidating the minimal requirements and physicochemical principles driving phase separation. For example, LLPS of proteins like FUS or TDP-43 has been reconstituted *in vitro* to model pathological condensate formation [118,119]. A primary limitation, however, is that these simplified systems lack the complexity of the intracellular environment, which may limit their ability to fully capture *in vivo* phenomena.

### 3.3 Optogenetics

Optogenetic approaches enable precise, real-time control of LLPS by using light to activate engineered proteins that undergo phase separation [120]. For instance, the optoDroplet platform utilizes light-inducible proteins fused to IDRs, such as FUS to drive condensate formation with high spatiotemporal precision upon light exposure [114,121]. This technique allows for fine-grained dissection of condensate assembly and disassembly. A significant constraint is its reliance on the expression of exogenous photosensitive constructs and the limited penetration of light, which can restrict its application in thick tissues or *in vivo* models.

### 3.4 Nuclear Magnetic Resonance (NMR) Spectroscopy

NMR spectroscopy provides molecular-level insights into the structural features and dynamics of biomolecules involved in LLPS [113]. By tracking the electromagnetic resonance of atomic nuclei, NMR can reveal conformational changes and interactions that occur during the phase separation process, particularly at high sample concentrations that promote condensation [122]. A key challenge is the method’s sensitivity to sample conditions, including concentration, temperature, and viscosity, which can confine its applicability to well-defined experimental systems.

### 3.5 Proteolysis-Targeting Chimeras (PROTACs)

PROTACs are bifunctional molecules that recruit a target protein to an E3 ubiquitin ligase, leading to its ubiquitination and degradation by the proteasome. This strategy can be used to disrupt LLPS by depleting key scaffold proteins [123]. For example, the PROTAC molecule ZXH-3-26 has been used to selectively degrade BRD4, enabling researchers to track the consequent changes in super-enhancer condensate dynamics [58]. While promising for interrogating the functional contribution of specific proteins to condensates, the effectiveness of PROTACs depends on the development of highly specific ligands and requires further optimization for *in vivo* applications.

### 3.6 Current LLPS Experimental Limitations

Despite the powerful insights provided by these techniques, the study of LLPS in a biological context faces several persistent challenges. First, the dynamic and reversible nature of LLPS makes it difficult to capture its rapid kinetics and microscopic heterogeneity in real time within living systems. Second, the biological relevance of *in vitro* models remains limited, as they often fail to recapitulate the complex biochemical and physical gradients found in the tumor microenvironment, such as variations in oxygen pressure and metabolite concentrations, which are known to influence LLPS. Third, quantitative analysis of key parameters such as condensate size, density, and phase transition thresholds often relies on semi-quantitative methods and lacks standardized metrics across studies. Finally, interventions targeting condensates, including small-molecule disruptors and PROTACs, may exhibit off-target effects that complicate the interpretation of results and pose potential toxicity concerns. To overcome these limitations, future methodological developments should focus on optimizing detection technologies such as super-resolution imaging, developing more physiologically relevant *in vitro* models, and establishing standardized quantitative analytical frameworks. Furthermore, the integration of proteomics, single-cell sequencing, and other multi-omics technologies with biophysical approaches promises to provide a more comprehensive understanding of LLPS in tumor biology and accelerate the clinical translation of targeted therapeutic strategies.

## 4 Therapeutic Strategies Targeting LLPS in Drug Resistance

The delineation of LLPS-mediated mechanisms in drug resistance naturally catalyzes the transition from basic research to therapeutic exploration. Traditional occupancy-based inhibitors often fail against dynamic condensates. Consequently, researchers are developing novel modalities to precisely target and disrupt these pro-survival assemblies (Table 2).

### 4.1 AI-Driven Design of IDR-Targeting Protein Binders

While techniques like FRAP and optogenetics are powerful for observing LLPS, they remain primarily diagnostic. A paradigm-shifting therapeutic advancement, however, originates from *de novo* protein design. The therapeutic frontier is now being reshaped by AI-powered platforms, such as RFdiffusion, which enable the design of high-affinity protein binders that specifically target IDRs [124]. This is pivotal because IDRs, the primary drivers of LLPS, were historically deemed “undruggable” due to their structural fluidity. This approach has successfully generated binders for diverse IDRs, including the Arabidopsis BRCA1 homolog (BRCA1_ARATH) [125]. These designed biologics hold the potential to inhibit pathological LLPS by sequestering key proteins or disrupting the multivalent interactions essential for phase separation [124,125], offering a direct means to disassemble resistance hubs.

### 4.2 Advancing Protein Degradation: From Single to Multi-Target PROTACs

PROTACs represent a promising tool to degrade specific oncoproteins like BRD4 and SPOP [58,123]. The field is now evolving to address the complexity of resistance networks through dual-/multi-target PROTACs [126,127]. These next-generation degraders are engineered to simultaneously recruit and degrade two or more disease-associated proteins. This strategy is particularly powerful against cancers where resistance is mediated by redundant pathways. For instance, a multi-target PROTAC could be designed to co-degrade a transcription factor within a drug-resistant condensate and a key pro-survival kinase, thereby delivering a more comprehensive therapeutic effect and overcoming the limitations of single-target inhibition [128].

### 4.3 Emerging Chemical Modalities: Covalent Bifunctional Molecules (CBMs)

Beyond biologics and degraders, covalent strategies offer a promising chemical avenue to tackle drug-resistant condensates. Covalent Bifunctional Molecules (CBMs) are designed to form stable, long-lasting bonds with specific target proteins, providing sustained pharmacodynamic effects [129]. Critically, recent studies demonstrate that covalent small molecules can be rationally designed to modify key nodes within biomolecular condensates. For instance, a charge-driven covalent modifier has been shown to inhibit stress granule formation by altering the conformation of G3BP1 [14]. Similarly, covalent ligands can induce conformational changes in the intrinsically disordered region of the androgen receptor, thereby modulating its phase separation behavior [130]. This approach aligns with the emerging paradigm of “condensate-modifying therapeutics” (c-mods) for cancer treatment [131]. Therefore, the application of CBM technology to key nodes within LLPS condensates could potentially lock them in a dysfunctional state, thereby abrogating their role in therapy resistance.

In summary, the therapeutic targeting of LLPS is rapidly advancing. The convergence of AI-based protein design, advanced degradation technologies, and covalent small molecules provides a versatile toolkit to disrupt the resilient condensates that fuel cancer drug resistance, offering new avenues for targeted oncology.

### 4.4 Challenges and Translational Considerations in LLPS-Targeted Therapies

While AI-driven IDR binders, multi-target PROTACs, and CBMs represent innovative modalities for disrupting oncogenic condensates [124,125,126,129], their clinical translation faces substantial hurdles. A primary challenge is selectivity: pathological condensates often share components with physiological ones such as stress granules or nucleoli, risking off-target disruption of essential cellular processes and systemic toxicity [131]. For instance, broad degradation via PROTACs may exacerbate this, as seen in ongoing clinical trials of non-LLPS-specific degraders, where dose-limiting toxicities have emerged [132].

Additionally, pharmacokinetic limitations hinder *in vivo* efficacy. Many condensate disruptors exhibit poor stability, rapid clearance, or inadequate tumor penetration, failing to achieve therapeutic concentrations at intracellular sites [131]. Preclinical models (primarily cell lines and xenografts) often overestimate efficacy by neglecting tumor microenvironment factors like hypoxia or stromal interactions, contributing to poor translation—similar to challenges in other condensate-targeted approaches [133]. To our knowledge, no dedicated LLPS-modulating agents have entered clinical trials, with promising preclinical strategies such as YAP condensate inhibitors stalling due to these gaps [134].

Contextual variability further complicates application: LLPS is highly sensitive to cellular conditions such as pH, crowding, leading to inconsistent effects across cancer subtypes or patients [111]. Monitoring therapeutic responses remains technically challenging, lacking robust biomarkers or real-time imaging for condensate dynamics in humans. These barriers explain the lag in clinical adoption despite preclinical promise. Future directions include developing selective “condensate-specific” ligands, nanoparticle delivery systems, and combination regimens with existing therapies to mitigate resistance. Addressing these will be crucial for realizing LLPS targeting as a viable anticancer strategy.

**Table 2:** Emerging therapeutic strategies targeting LLPS in drug resistance.

Therapeutic Modality	Molecular Target	Mechanism of Action	Stage	Reference
AI-Designed Protein Binders	IDRs of oncoproteins	Disrupt multivalent interactions and prevent pathological condensate assembly by sequestering key scaffold proteins.	Preclinical	[124,125]
PROTACs	Scaffold proteins in condensates	Induce targeted degradation of key condensate components, disassembling oncogenic hubs.	Preclinical	[58,123,126,127,128]
CBMs	Critical interfaces within condensates	Form stable, covalent bonds to lock targets in a dysfunctional state, potentially abrogating condensate function.	Theoretical/Early Research	[14,129,130,131]
Small Molecule Inducers	β-catenin	Promote LLPS that contributes to target degradation	Preclinical	[65]
Repurposed Agents	PrP	Disrupt PrP LLPS to reduce drug efflux and ROS, restoring chemosensitivity.	Preclinical	[64]

Note: LLPS: Liquid-liquid phase separation; AI: Artificial Intelligence; IDRs: intrinsically disordered regions; PROTACs: Proteolysis-Targeting Chimeras; CBMs: Covalent Bifunctional Molecules; PrP: prion protein; ROS: reactive oxygen species.

## 5 Summary and Perspective

This review synthesizes evidence that LLPS drives cancer drug resistance through the formation of functional biomolecular condensates. We have framed LLPS not merely as a collection of discrete mechanisms, but as a central adaptive hub that coordinates a network of survival responses to therapy. These dynamic assemblies facilitate a multitude of pro-survival mechanisms, including the establishment of transcriptional hubs that sustain oncogenic expression and the creation of physical barriers that limit drug penetration. They also enhance DNA repair efficiency and sustain cancer stemness. Together, these mechanisms enable tumor to evade various therapeutic modalities. The interplay between LLPS and key oncogenic signaling pathways, such as Wnt, Hippo, and TGF-β, further enhances these adaptive pro-survival responses. Moreover, LLPS regulates fundamental cellular dynamic processes, including cell cycle progression, microtubule stability, and apoptotic signaling. This establishes a cohesive framework that underpins tumor resilience. Targeting these LLPS-mediated processes offers a promising avenue for developing next-generation anticancer strategies. Despite increasing awareness, translating LLPS biology into clinical applications faces several challenges. A primary gap is the inability to dynamically capture LLPS kinetics and heterogeneity in living tumor. Future research should prioritize the development of advanced real-time imaging technologies and more physiologically relevant models, such as tumor organoids, to decipher LLPS dynamics within the native tumor microenvironment. Furthermore, the contextual regulation of LLPS by metabolic and immune factors remains poorly characterized. Leveraging CRISPR-based screens and multi-omics profiling will be essential to identify key genetic and biophysical regulators of pathological phase separation. These efforts will provide the foundation for novel therapeutic interventions, which may include small-molecule inhibitors targeting critical condensate components, PROTACs designed for specific degradation of scaffold proteins, and combinatorial strategies that integrate LLPS disruption with established modalities like immunotherapy. The field is now approaching a pivotal juncture. The convergence of biophysical insights, AI-driven drug design, and functional genomics promises to accelerate the development of ‘condensate medicine’ rapidly. As these tools mature, the strategic disruption of oncogenic condensates holds exceptional potential to overcome the pervasive challenge of drug resistance, ushering in a new era of targeted and personalized cancer therapeutics.

## Data Availability

Not applicable.

## Abbreviations

LLPS	Liquid-liquid phase separation
IDRs	intrinsically disordered regions
CRC	colorectal cancer
Dvl2	Dishevelled 2
lncRNAs	Long non-coding RNAs
HCC	hepatocellular carcinoma
HRR	homologous recombination repair
PROTACs	Proteolysis-Targeting Chimeras
HK	hexokinase
PKA	protein kinase A
MDR	multidrug resistance
PrP	prion protein
ROS	reactive oxygen species
HNSCC	head and neck squamous cell carcinoma
DDR	DNA Damage Response
APA	alternative polyadenylation
ZO	zonula occludens
NuMA	nuclear mitotic apparatus protein
APL	acute promyelocytic leukemia
CSCs	cancer stem cells
CycT1	Cyclin T1
P-TEFb	positive transcription elongation factor b
EMT	Epithelial-Mesenchymal Transition
NMR	nuclear magnetic resonance
CBMs	Covalent Bifunctional Molecules
c-mods	condensate-modifying therapeutics
